# Multicolor tunable bright photoluminescence in Ca^2+^/Mg^2+^ modified Eu^3+^ doped ZnGa_2_O_4_ phosphors under UV excitation for solid state lighting applications

**DOI:** 10.1039/d3ra03215a

**Published:** 2023-07-05

**Authors:** Monika, R. S. Yadav, A. Bahadur, Shyam Bahadur Rai

**Affiliations:** a Laser & Spectroscopy Laboratory, Department of Physics, Institute of Science, Banaras Hindu University Varanasi 221005 India sbrai49@yahoo.co.in; b Department of Zoology, Institute of Science, Banaras Hindu University Varanasi 221005 India

## Abstract

The Eu^3+^ doped and Mg^2+^/Ca^2+^ co-doped ZnGa_2_O_4_ phosphor samples were synthesized by solid-state reaction method and their structural and optical properties studied. The phase, crystallinity and particles size of the phosphor samples were studied by XRD and SEM measurements. EDS analyses were used to identify the elements present in the phosphor materials. The vibrational groups present in the phosphor samples were examined by Fourier transform infrared (FTIR) measurements. Pure ZnGa_2_O_4_ emits intense blue light under 260 nm excitation. However, Eu^3+^ doped and Mg^2+^/Ca^2+^ co-doped ZnGa_2_O_4_ phosphor samples exhibit intense red emission under 393 nm excitation. A bluish white color is observed in these samples under 290 nm excitation. The maximum PL emission intensity is found at 0.1 mol% Eu^3+^ doping concentration. For higher concentrations, concentration quenching was observed due to dipole–dipole interaction. The emission intensity is enhanced upto 1.20 and 2.91 times on co-doping of Mg^2+^ and Ca^2+^*via* induced crystal field due to charge imbalance. The emission intensity of the phosphor is found to enhance further on annealing the samples at 873 K. Under various excitation wavelengths, color tunability was seen from blue to bluish-white to red regions. The lifetime of the ^5^D_0_ level of the Eu^3+^ ion improves *via* doping of Mg^2+^/Ca^2+^ ions and it increases appreciably on annealing. The temperature dependent photoluminescence study (TDPL) reveals a thermal quenching behavior of the sample with thermal stability ∼65% and activation energy ∼0.223 eV in the Eu^3+^/Ca^2+^ co-doped ZnGa_2_O_4_ phosphor sample.

## Abstract 2

The Eu^3+^/Mg^2+^/Ca^2+^ doped and co-doped ZnGa_2_O_4_ phosphors give blue and red emissions, which are enhanced *via* Mg^2+^/Ca^2+^ doping. They also show better color tunability and thermal stability.

## Graphical abstract

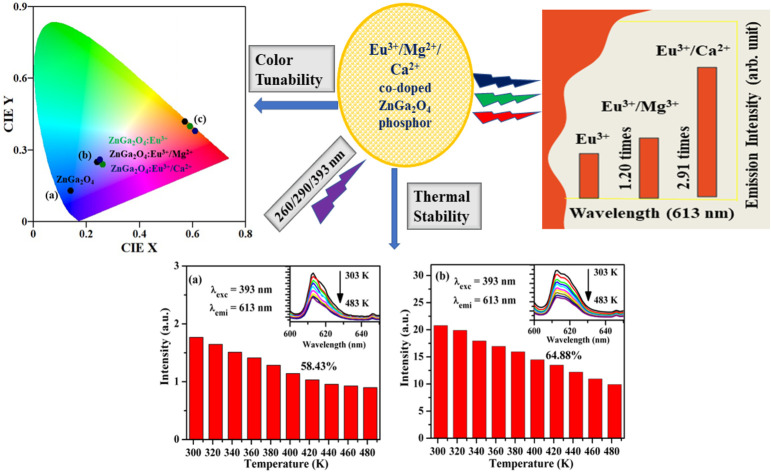

## 1. Introduction

The rare earth doped inorganic phosphor materials are highly chemically, physically and thermally stable and yield large photoluminescence intensity on excitation with suitable wavelength. These materials have wide applications in different emerging fields, such as display devices, light emitting diodes (LEDs), color tunable devices, temperature sensing, development of new lasers, plant cultivation *etc.*^1–7^ This is possible due to the presence of a large number of meta-stable energy levels in the rare earth ions.^8–12^ The rare earth ions, such as Eu^3+^, Tb^3+^, Tm^3+^, Dy^3+^, *etc.*, emit red, green, blue and yellow colors respectively, in different host matrices.^2,5–7^ Thus, a combination of these rare earth ions, such as Dy^3+^/Eu^3+^, Sm^3+^/Eu^3+^, Tb^3+^/Eu^3+^, *etc.* produces color tunable photoluminescence (PL) in different host matrices depending on their concentrations and excitation wavelengths.^13–16^ The concentration of these ions plays a very important role in achieving color tunable emissions. This occurs due to a variation in the intensity of the emitted colors from these ions as well as energy transfer between them. Out of these rare earth ions, the Eu^3+^ ion is very promising as it gives almost pure red emission.^17–20^ The Eu^3+^ doped phosphor materials are often used to fulfill the requirement of red components in white LEDs. However, the PL intensity of Eu^3+^ doped phosphor materials needs to be improved.

Efforts have been made by several groups and it is still required to enhance the PL emission intensity of Eu^3+^ ion in different hosts by co-doping the alkalis, alkaline earths, transition metals and rare earth ions.^21–26^ In these cases, the PL intensity of Eu^3+^ ion is enhanced not only due to increase in crystallinity of the materials but also due to crystal field effect of the other doped ions and energy transfer to Eu^3+^ ion by other ions.^13–16,21–26^ Yang *et al.* have prepared the novel red-emitting Sr_7_Sb_2_O_12_:Eu^3+^, M^+^ (M = Li, Na, K) phosphors and studied the effect of alkali ions on the PL intensity of Eu^3+^.^21^ Our group has also studied the impact of alkali doping on the PL intensity of Eu^3+^ ions in CaTiO_3_ phosphor.^22^ Singh *et al.* have reported luminescent characteristics of M_3_Y_2_Si_3_O_12_:Eu^3+^ (M = Ca, Mg, Sr and Ba) and found significant enhancement in the PL emission intensity in presence of these ions.^23^ Shi *et al.* have reported an enhancement in the PL intensity of Eu^3+^ ion in Y_2_O_3_:Eu^3+^ phosphors in presence of alkali and alkaline earth metal ions.^27^ The enhancement in PL intensity has been also observed due to charge compensation (crystal field effect), increase in crystallinity and asymmetric nature of the crystal field. Yang *et al.* have also observed that increasing the concentration of Bi^3+^ ions led to an improvement in the PL intensity of Eu^3+^ in ZnGa_2_O_4_ phosphor, which is caused by energy transfer from Bi^3+^ to Eu^3+^ ions.^28^ Rai *et al.* have observed enhancement in PL intensity of Eu^3+^ ion through energy transfer from Tb^3+^ to Eu^3+^ ions in LaVO_4_ phosphor.^16^ However, the effect of co-doping of Mg^2+^ and Ca^2+^ ions on the PL intensity in ZnGa_2_O_4_:Eu^3+^ phosphor has not been investigated to our knowledge. Our group has found that the PL intensity of LaVO_4_:Eu^3+^ phosphor was enhanced 4.5 times *via* co-doping of Ca^2+^ ion.^24^ In the present work, the PL intensity of Eu^3+^ doped ZnGa_2_O_4_ phosphor has been investigated in absence and presence of Mg^2+^/Ca^2+^ ions.

The thermal stability of phosphor material is one of the desirable conditions for practical applications as it is an important parameter for a photoluminescent phosphor. The variation of PL emission intensity with temperature is a function of thermal stability of the phosphor materials.^29^ The thermal stability of phosphor samples are compared in terms of photoluminescence emission at 423 K (150 °C) for LEDs applications as the phosphor materials deteriorate at higher temperatures and reduce its emission efficiency.^30^ The temperature dependent PL intensity has been studied by Rajendran *et al.* in Ba_2_YV_3_O_11_:Eu^3+^ phosphor and found the thermal stability of phosphor is 59.5% at 423 K.^31^ In the case of Ba_2_LaV_3_O_11_:Eu^3+^, this value was reported to be 62% at 423 K.^32^ The temperature-dependent PL in the Bi_4_Si_3_O_12_:Eu^3+^ phosphor was also studied by Zhang *et al.*^33^ They have found that the PL emission intensity is decreased to 50% at 398 K compared to its PL intensity at 298 K. It would be interesting to measure the thermal stability of Eu^3+^ doped and Eu^3+^/Ca^2+^ co-doped ZnGa_2_O_4_ phosphor material.

In this work, the Eu^3+^ doped and Mg^2+^/Ca^2+^ co-doped ZnGa_2_O_4_ phosphor materials have been synthesized through solid state reaction method at 1473 K. A small part of the prepared samples has been annealed at 873 K temperature to see the changes in structural and photoluminescence properties of the doped and co-doped samples. The X-ray diffraction (XRD), scanning electron microscopic (SEM) and energy dispersive X-ray spectroscopic (EDS) measurements have been carried out for the structural, morphological and elemental properties. The vibrational structures of the phosphor samples have been studied by Fourier transform infrared (FTIR) measurements. The Eu^3+^ doped ZnGa_2_O_4_ phosphor sample emits bright red color along with blue color on excitation with charge transfer band (CTB) of host at 260 nm and the charge transfer band (CTB) of Eu^3+^ at 290 nm. However, on excitation with *n*-UV wavelength at 393 nm (atomic line of Eu^3+^), only red emission is seen due to Eu^3+^ ion. The PL intensity of Eu^3+^ doped phosphor is enhanced on co-doping of Mg^2+^/Ca^2+^ ions. On annealing the samples at 873 K, the PL intensity of phosphor samples was further improved. The CIE coordinates of the phosphor samples were calculated for undoped and doped samples. The lifetime of ^5^D_0_ level of Eu^3+^ ion has been measured using ^5^D_0_ → ^7^F_2_ transition at 613 nm wavelength under the excitation with 393 nm. The thermal stability of the Eu^3+^ doped and Eu^3+^/Ca^2+^ co-doped ZnGa_2_O_4_ phosphor samples were demonstrated by the temperature dependent photoluminescence (TDPL) studies. These values in the two cases were found to be 58.43% and 64.88% with activation energies 0.198 eV and 0.223 eV, respectively at 423 K.

## 2. Experimental methods

### 2.1 Synthesis

The phosphor samples have been synthesized by a solid-state reaction method at 1473 K temperature. We have used base materials as ZnO (Otto, 99.99%), Ga_2_O_3_ (Alfa Aesar 99.9%), Eu_2_O_3_ (Molychem, 99.99%), MgO (Himedia, 99.9%) and CaCO_3_ (SDFCL, 99%). These materials were weighed carefully followed by mixing in agate mortar and acetone as mixing medium. The homogenously mixed samples were put in alumina crucible and heated in an electric furnace at 1473 K temperature for 4 hours. These samples were crushed into fine powder. A small part of these samples were further annealed at 873 K temperature for 4 hours separately to see the effect of further heating. Following compositions were used for the sample preparation:

i 47ZnO + 53Ga_2_O_3_ → 47(ZnGa_2_O_4_) + 6Ga_2_O_3_

ii 47ZnO + (53 − *x*)Ga_2_O_3_ + *x*Eu_2_O_3_ → 47(ZnGa_2−*x*_O_4+*δ*_):*x*Eu + 6Ga_2_O_3_

where *x* was taken (0.05, 0.1, 0.2, 0.5 and 1.0 mol%) the concentration of Eu^3+^, respectively. In Mg^2+^/Ca^2+^ co-doped ZnGa_2_O_4_:Eu^3+^ phosphor samples, the compositions used were as follows:

iii 47ZnO + (53 − *x* − *y*)Ga_2_O_3_ + *x*Eu_2_O_3_ + *y*MgO → 47(ZnGa_2−*x*−*y*_O_4+*δ*_):*x*Eu:*y*Mg + 6Ga_2_O_3_

iv 47ZnO + (53 − *x* − *z*)Ga_2_O_3_ + *x*Eu_2_O_3_ + *z*CaCO_3_ → 47(ZnGa_2−*x*−*z*_O_4+*δ*_):*x*Eu:*z*Ca + 6Ga_2_O_3_ + CO_2_

where *x* was fixed at 0.1 mol% concentration and the *y* and *z* were varied as 1, 2, 3 and 5 mol% concentrations to get the optimum PL intensity. The term ‘*δ*’ represents the excess of oxygen released during the synthesis. These phosphor samples were used for further analyses.

### 2.2 Instrumentation

The crystalline nature and phase purity of the phosphor samples were analyzed by monitoring the XRD patterns using Rigaku diffractometer (MiniFlex 600-unit and Cu Kα radiation with *λ* = 0.15406 nm). The scanning electron microscope (SEM) (Zeiss, Evo 18 Research unit) was used to study the morphological structure of the phosphor samples. The elements present in the phosphors were verified by the energy dispersive X-ray spectroscopic (EDS) studies. The Fourier transform infrared (FTIR) spectra were monitored to know the vibrational groups present in the phosphors using a PerkinElmer IR spectrometer (I-Frontier unit). The downshifting photoluminescence spectra of all the samples were recorded using Fluorolog-3 spectrophotometer (Horiba Jobin Yvon) attached with a 450 W Xenon lamp as a source of light (Horiba Jobin Yvon). We have also measured the lifetime of ^5^D_0_ level of Eu^3+^ ion using the same unit attached with a 25 W pulsed Xenon lamp.

## 3. Results and discussion

### 3.1 Structural studies

#### 3.1.1 X-ray diffraction (XRD) measurements

**Fig. 1:**
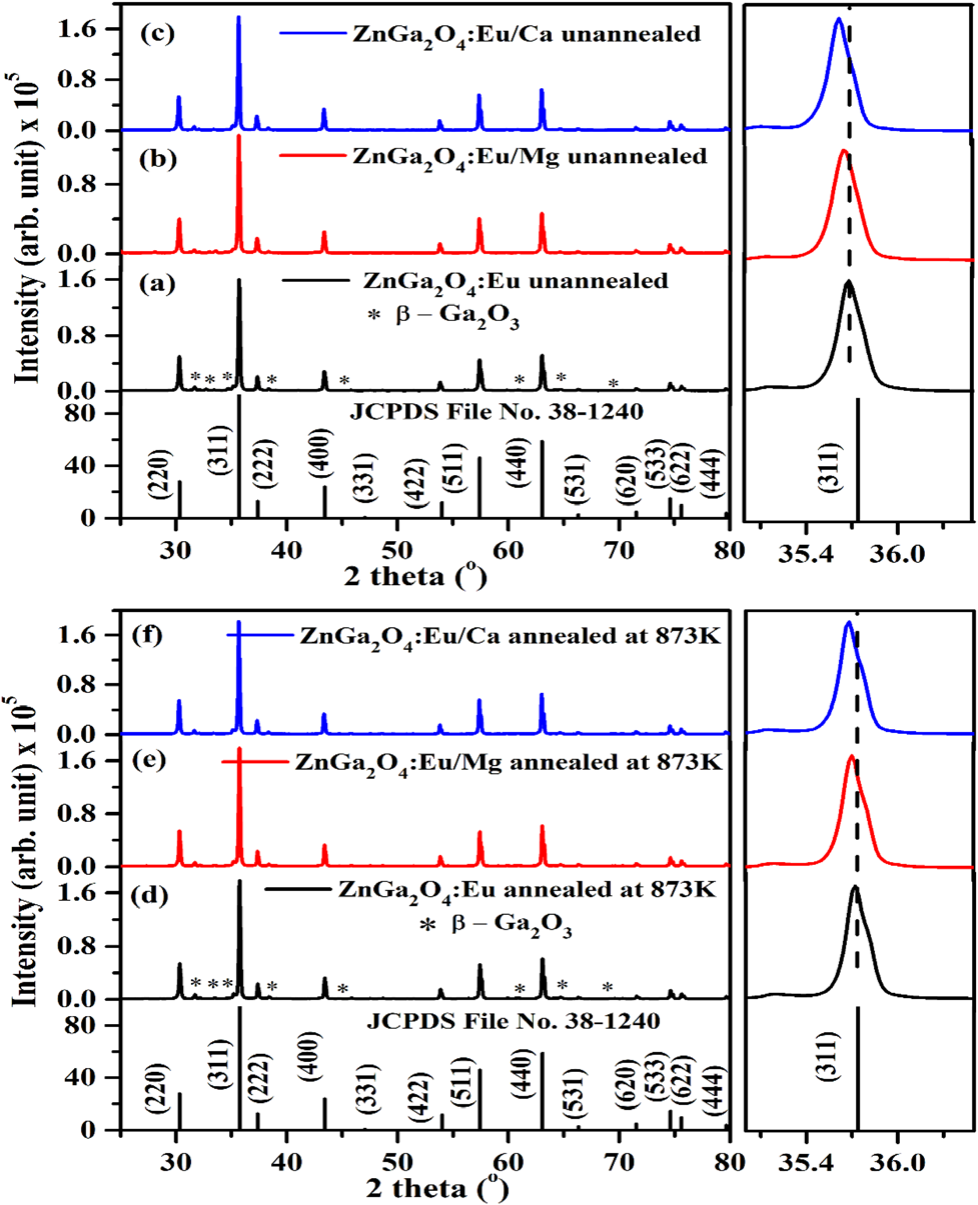
XRD patterns of (a) ZnGa_2_O_4_:0.1Eu^3+^ (b) ZnGa_2_O_4_:0.1Eu^3+^/3Mg^2+^ and (c) ZnGa_2_O_4_:0.1Eu^3+^/3Ca^2+^ phosphor samples and that of (d) ZnGa_2_O_4_:0.1Eu^3+^ (e) ZnGa_2_O_4_:0.1Eu^3+^/3Mg^2+^ and (f) ZnGa_2_O_4_:0.1Eu^3+^/3Ca^2+^ phosphor samples annealed at 873 K along with JCPDS File No. and their zoomed patterns of (311) lattice plane.

The XRD patterns of ZnGa_2_O_4_:0.1Eu^3+^, ZnGa_2_O_4_:0.1Eu^3+^/3Mg^2+^ and ZnGa_2_O_4_:0.1Eu^3+^/3Ca^2+^ phosphor samples unannealed and annealed (at 873 K) are shown in Fig. 1(a–f). The phase of the spinel crystal is cubic. The diffraction peaks match well with standard JCPDS File No. 38-1240.^2,4–7,35,36^ Some weak impurity peaks were also observed in the XRD patterns due to monoclinic phase of β-Ga_2_O_3_. These impurity peaks are marked with asterisk ‘*’ in Fig. 1. The sharpness of the diffraction peaks indicates highly crystalline nature of the prepared phosphor. When the Mg^2+^/Ca^2+^ ions are co-doped in the ZnGa_2_O_4_:0.1Eu^3+^ phosphor at Ga^3+^ site, the XRD peaks are shifted towards lower 2*θ* angle side. The peaks are shifted as the ionic radii of Ca^2+^ (100 pm) and Mg^2+^ (72 pm) are higher as compared to Ga^3+^ (62 pm) ion.^23^ The shift in XRD peak position can be verified from the zoomed patterns shown in Fig. 1(a–f).

The phosphor samples annealed at 873 K temperature show an improvement in crystallinity of the materials. The average crystallite size (*D*) were calculated using Debye Scherrer's formula.^34–37^v
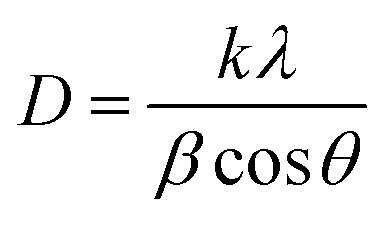
where *k* is a constant (=0.89), *β* is the full width at half maxima (FWHM) at a diffraction angle (*θ*) and *λ* is the wavelength of X-ray radiation. The obtained values of crystallite size are found to be 38.09, 39.06 and 41.27 nm for the ZnGa_2_O_4_:0.1Eu^3+^, ZnGa_2_O_4_:0.1Eu^3+^/3Mg^2+^ and ZnGa_2_O_4_:0.1Eu^3+^/3Ca^2+^ phosphor samples, respectively. This shows that the crystallite size increases on co-doping of Ca^2+^/Mg^2+^ ions. The crystallite size is further improved on annealing the samples at 873 K and the values were found to be 41.29, 42.15 and 43.19 nm, respectively. The increase in crystallite size would be supportive for enhancing the PL intensity of the phosphor samples.

We have also analyzed the dislocation density for the ZnGa_2_O_4_:0.1Eu^3+^, ZnGa_2_O_4_:0.1Eu^3+^/3Mg^2+^ and ZnGa_2_O_4_:0.1Eu^3+^/3Ca^2+^ phosphor samples and the samples annealed at 873 K by using the following relation:^7,35,36^vi
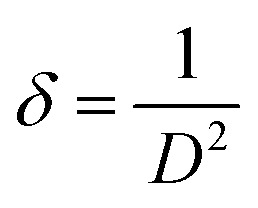


The computed dislocation densities for the ZnGa_2_O_4_:0.1Eu^3+^, ZnGa_2_O_4_:0.1Eu^3+^/3Mg^2+^ and ZnGa_2_O_4_:0.1Eu^3+^/3Ca^2+^ phosphor samples are 6.8 × 10^−4^, 6.5 × 10^−4^ and 5.8 × 10^−4^ nm^−2^, respectively. In the case of annealed samples these values are 5.8 × 10^−4^, 5.6 × 10^−4^ and 5.3 × 10^−4^ nm^−2^, respectively. This shows that the dislocation density decreases in presence of Mg^2+^/Ca^2+^ ions thereby improve the local crystal structure, which is responsible for the enhancement of PL intensity of the phosphor materials.

**Fig. 2:**
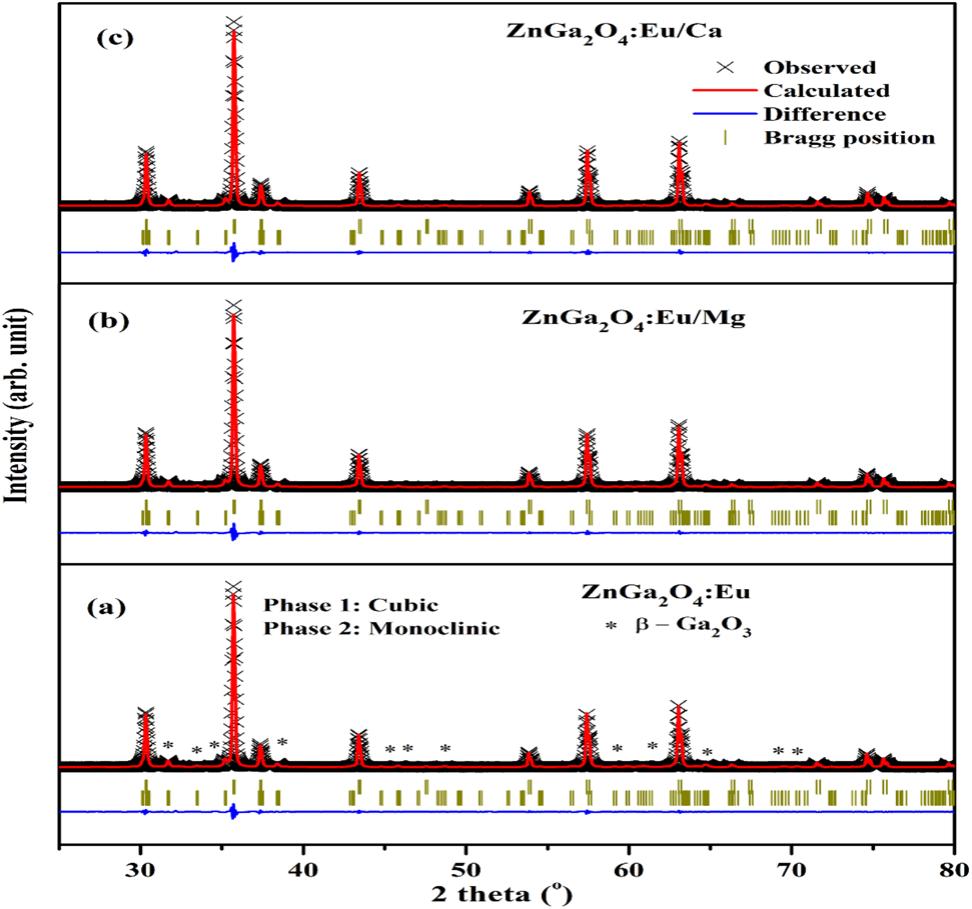
Rietveld fits of XRD patterns for (a) ZnGa_2_O_4_:0.1Eu^3+^ (b) ZnGa_2_O_4_:0.1Eu^3+^/3Mg^2+^ and (c) ZnGa_2_O_4_:0.1Eu^3+^/3Ca^2+^ phosphor. Asterisks ‘*’ represents the impurity peaks due to β-Ga_2_O_3_.

The Rietveld refinements of XRD patterns for the ZnGa_2_O_4_:0.1Eu^3+^, ZnGa_2_O_4_:0.1Eu^3+^/3Mg^2+^ and ZnGa_2_O_4_:0.1Eu^3+^/3Ca^2+^ phosphor samples have been carried out using the FullProf program and they are shown in Fig. 2(a–c). The Fig. 2(a–c) shows that the observed and calculated XRD patterns match well with each other. The lower profile represents the difference between the observed and the calculated XRD patterns, whereas the vertical bars are Bragg's positions of ZnGa_2_O_4_ (cubic) and β-Ga_2_O_3_ (monoclinic) phases. The different crystallographic parameters, such as phase, space group, lattice parameters and unit cell volumes for the ZnGa_2_O_4_:0.1Eu^3+^, ZnGa_2_O_4_:0.1Eu^3+^/3Mg^2+^ and ZnGa_2_O_4_:0.1Eu^3+^/3Ca^2+^ phosphor samples are summarized in Table 1.

**Table 1:** Phase, space group, lattice parameters and unit cell volume for the ZnGa_2_O_4_:0.1Eu^3+^, ZnGa_2_O_4_:0.1Eu^3+^/3Mg^2+^ and ZnGa_2_O_4_:0.1Eu^3+^/3Ca^2+^ phosphor

Sample	Phase (1)	Space group	Lattice parameters (ZnGa_2_O_4_)	Volume (Å^3^)	Phase (2)	Space group	Lattice parameters (β-Ga_2_O_3_)	Volume (Å^3^)
ZnGa_2_O_4_:0.1Eu^3+^	Cubic	*Fd*3̄*m*	*a* = 8.3311 Å	578.24	Monoclinic	*C*2/*m*	*a* = 12.216 Å	209.38
			*b* = 8.3311 Å				*b* = 3.0395 Å	
			*c* = 8.3311 Å				*c* = 5.8084 Å	
			*α* = *β* = *γ* = 90°				*α* = 90°	
							*β* = 103.876°	
							*γ* = 90°	
ZnGa_2_O_4_:0.1Eu^3+^/3Mg^2+^	Cubic	*Fd*3̄*m*	*a* = 8.3328 Å	578.60	Monoclinic	*C*2/*m*	*a* = 12.222 Å	209.51
			*b* = 8.3328 Å				*b* = 3.0389 Å	
			*c* = 8.3328 Å				*c* = 5.8101 Å	
			*α* = *β* = *γ* = 90°				*α* = 90°	
							*β* = 103.865°	
							*γ* = 90°	
ZnGa_2_O_4_:0.1Eu^3+^/3Ca^2+^	Cubic	*Fd*3̄*m*	*a* = 8.3339 Å	578.81	Monoclinic	*C*2/*m*	*a* = 12.222 Å	209.63
			*b* = 8.3339 Å				*b* = 3.0398 Å	
			*c* = 8.3339 Å				*c* = 5.8117 Å	
			*α* = *β* = *γ* = 90°				*α* = 90°	
							*β* = 103.861°	
							*γ* = 90°	

#### 3.1.2 SEM and EDS measurements

**Fig. 3:**
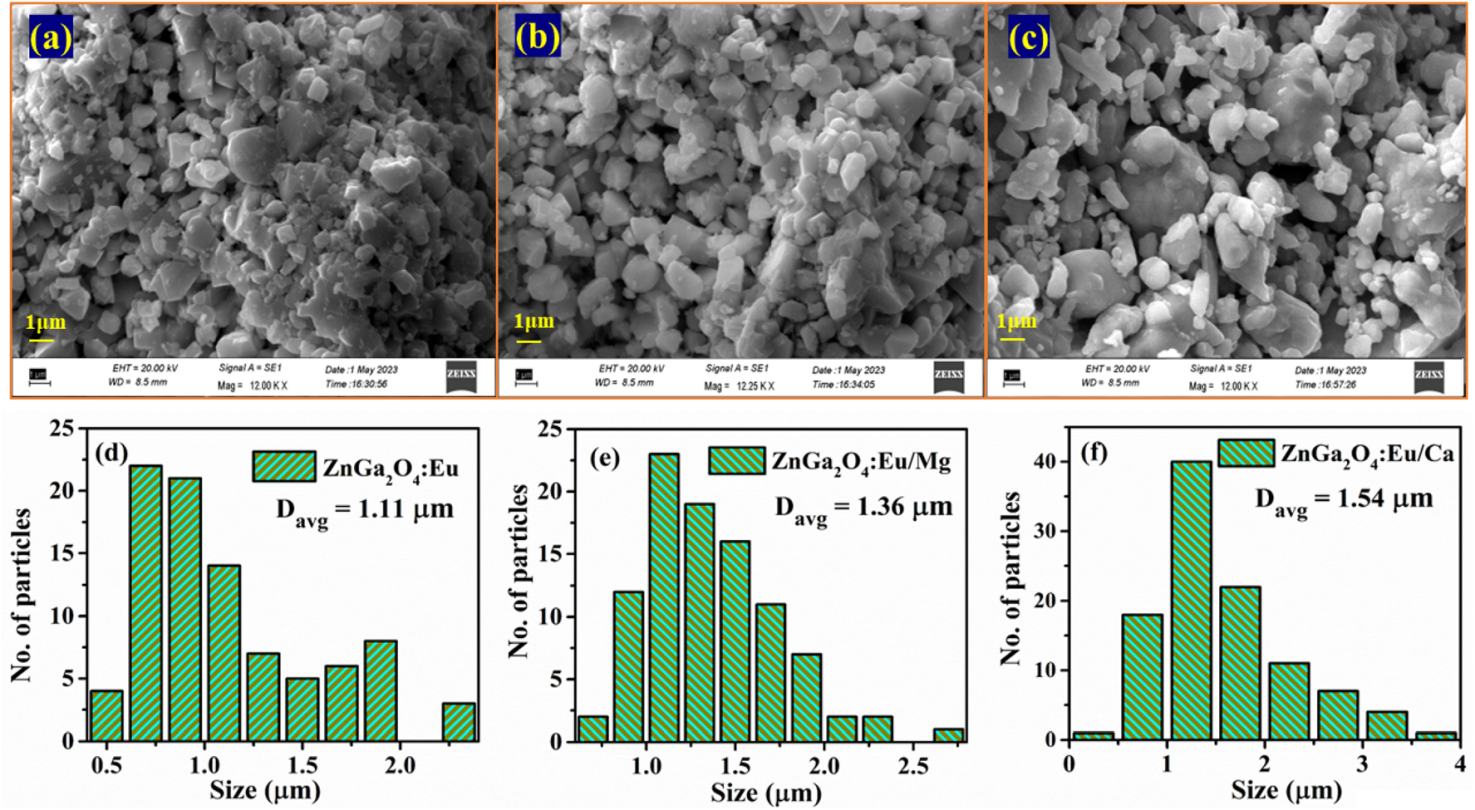
SEM images of (a) ZnGa_2_O_4_:0.1Eu^3+^ (b) ZnGa_2_O_4_:0.1Eu^3+^/3Mg^2+^ and (c) ZnGa_2_O_4_:0.1Eu^3+^/3Ca^2+^ and particles size distribution in (d) ZnGa_2_O_4_:0.1Eu^3+^ (e) ZnGa_2_O_4_:0.1Eu^3+^/3Mg^2+^ and (f) ZnGa_2_O_4_:0.1Eu^3+^/3Ca^2+^ phosphors.

The SEM images of ZnGa_2_O_4_:0.1Eu^3+^, ZnGa_2_O_4_:0.1Eu^3+^/3Mg^2+^ and ZnGa_2_O_4_:0.1Eu^3+^/3Ca^2+^ phosphor samples are shown in Fig. 3(a–c). It is clear from the figure that the particles shape is nearly spherical and agglomerated to each other. The particles size distribution has been evaluated by plotting histogram for the different phosphor samples using ImageJ software and they are shown in Fig. 3(d–f). The average particles size is found to be 1.11 μm for ZnGa_2_O_4_:0.1Eu^3+^ phosphor, which is slightly increased on doping of Mg^2+^ (1.36 μm) and Ca^2+^ (1.54 μm) ions in the ZnGa_2_O_4_:Eu^3+^ phosphor, respectively.^38^ This clearly shows that the average particles size of the phosphor materials are increased in presence of Mg^2+^/Ca^2+^ ions. Maurya *et al.* have also observed an increase in particles size of the Ho^3+^/Yb^3+^ co-doped CaZrO_3_ phosphor after co-doping of Mg^2+^ ions and reported an enhancement in the emission intensity.^38^ An increase in the particles size of phosphor has been also reported by Rai *et al.* in the LaVO_4_:Eu^3+^ phosphor on incorporation of Ca^2+^ ion.^24^ They have also observed an increase in the PL intensity of phosphor *via* Ca^2+^ doping.

**Fig. 4:**
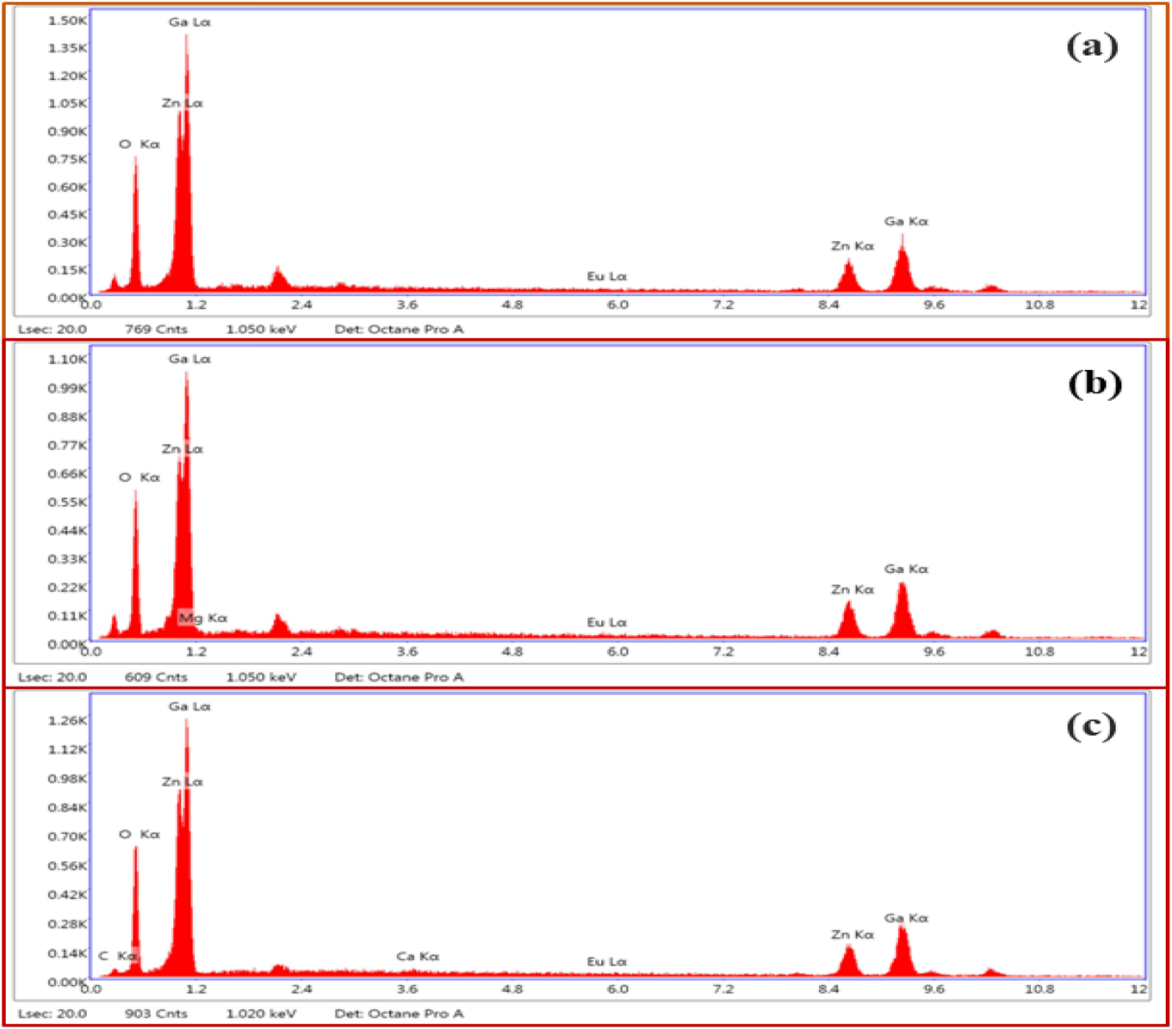
EDS spectra of (a) ZnGa_2_O_4_:0.1Eu^3+^ (b) ZnGa_2_O_4_:0.1Eu^3+^/3Mg^2+^ and (c) ZnGa_2_O_4_:0.1Eu^3+^/3Ca^2+^ phosphors.

Fig. 4(a–c) shows the energy dispersive X-ray spectroscopic (EDS) spectra of ZnGa_2_O_4_:0.1Eu^3+^, ZnGa_2_O_4_:0.1Eu^3+^/3Mg^2+^ and ZnGa_2_O_4_:0.1Eu^3+^/3Ca^2+^ phosphor samples, respectively. Fig. 4(a) clearly shows the presence of Zn, Ga, Eu and O elements in the phosphor samples. However, the incorporation of Mg and Ca elements along with these elements can be also verified by Fig. 4(b and c).

### 3.2 Optical measurements

#### 3.2.1 Fourier transform infrared (FTIR) measurements

**Fig. 5:**
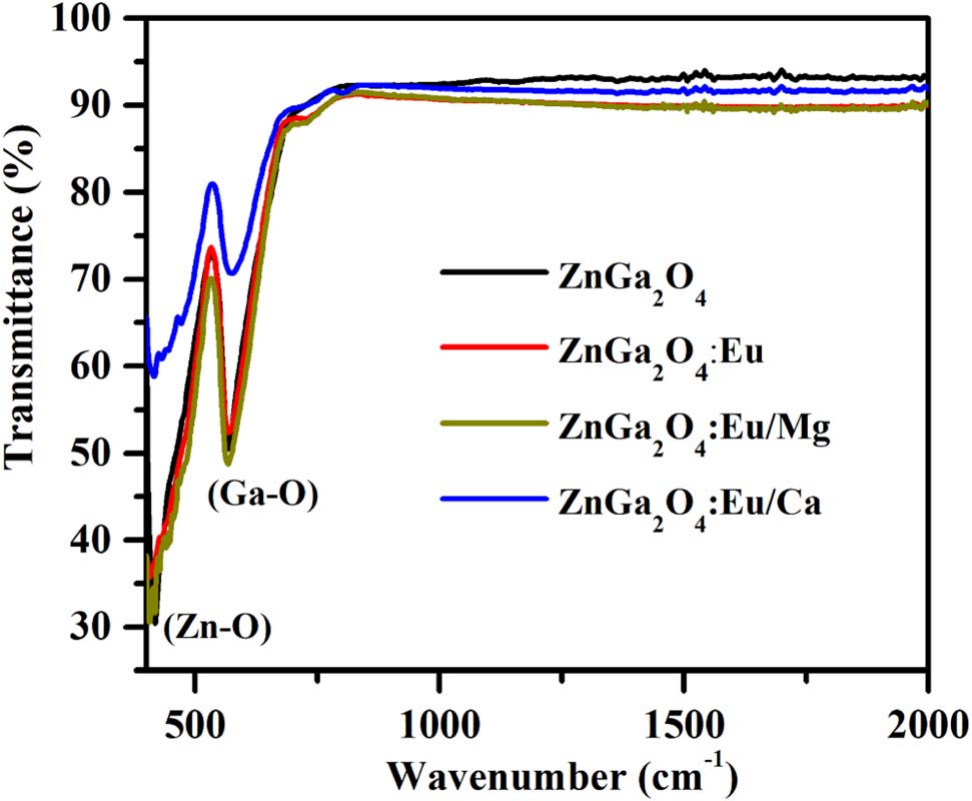
FTIR spectra of ZnGa_2_O_4_, ZnGa_2_O_4_:0.1Eu^3+^, ZnGa_2_O_4_:0.1Eu^3+^/3Mg^2+^ and ZnGa_2_O_4_:0.1Eu^3+^/3Ca^2+^ phosphor samples.

FTIR spectra of ZnGa_2_O_4_, ZnGa_2_O_4_:0.1Eu^3+^, ZnGa_2_O_4_:0.1Eu^3+^/3Mg^2+^ and ZnGa_2_O_4_:0.1Eu^3+^/3Ca^2+^ phosphor samples were recorded in the 400–2000 cm^−1^ range and they are shown in Fig. 5. The spectra show two vibrational bands at 419 and 569 cm^−1^ and they are assigned to arise due to stretching vibrations of the Zn–O (419 cm^−1^) and Ga–O (569 cm^−1^) groups, respectively.^7,35,36^ No impurity peaks are seen in the phosphor samples. It has been observed that there is no change in vibrational band's positions however, the absorption intensity of the Zn–O and Ga–O bands are found to increase due to doping of Eu^3+^, Ca^2+^ and Mg^2+^ ions.

#### 3.2.2 PL excitation (PLE) and PL emission measurements

**Fig. 6:**
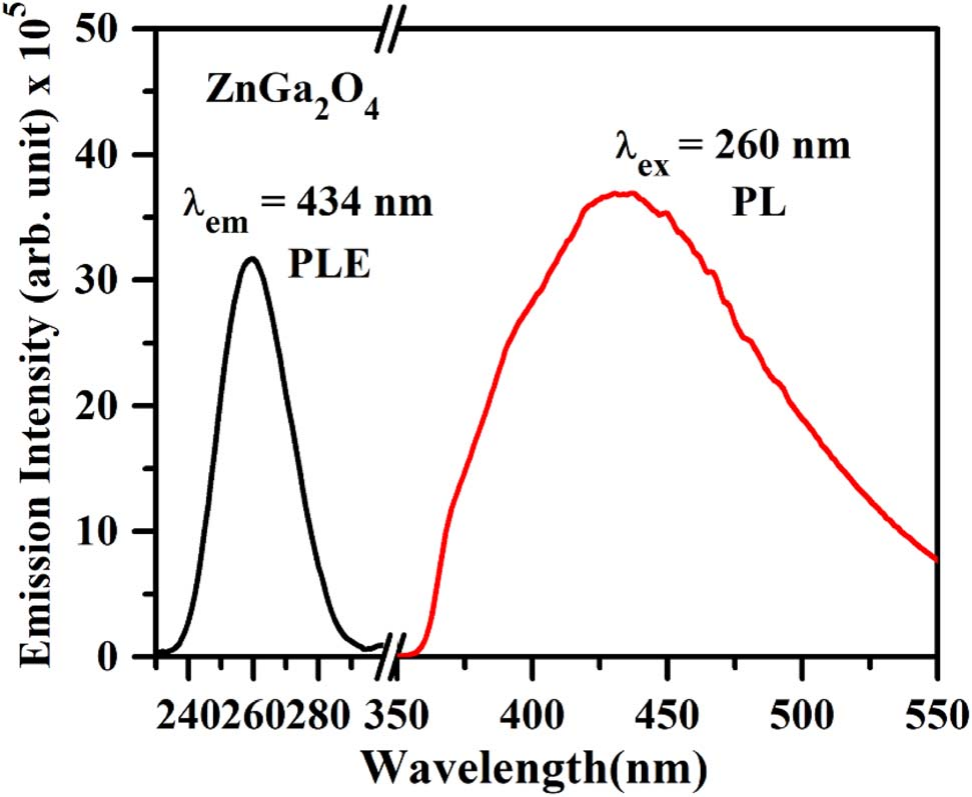
PL excitation spectrum of ZnGa_2_O_4_ under *λ*_em_ = 434 nm and PL emission spectrum of ZnGa_2_O_4_ phosphor under *λ*_ex_ = 260 nm.

The photoluminescence excitation (PLE) spectrum of ZnGa_2_O_4_ corresponding to *λ*_em_ = 434 nm and the photoluminescence (PL) emission spectrum under 260 nm excitation are shown in Fig. 6. The 260 nm band is due to charge transfer band of O^2−^ → Ga^3+^. Upon 260 nm excitation, the emission spectrum of the ZnGa_2_O_4_ sample emits a broad intense band ranging from 350 to 550 nm with the peak position maxima at 434 nm, which can be attributed to the self-activation center of the octahedral Ga–O group in the spinel lattice.^1,4^ The emission of this self-activated phosphor sample matches well with the absorption spectrum of plant pigments, which shows usefulness of this material for plant growth applications.^39^

**Fig. 7:**
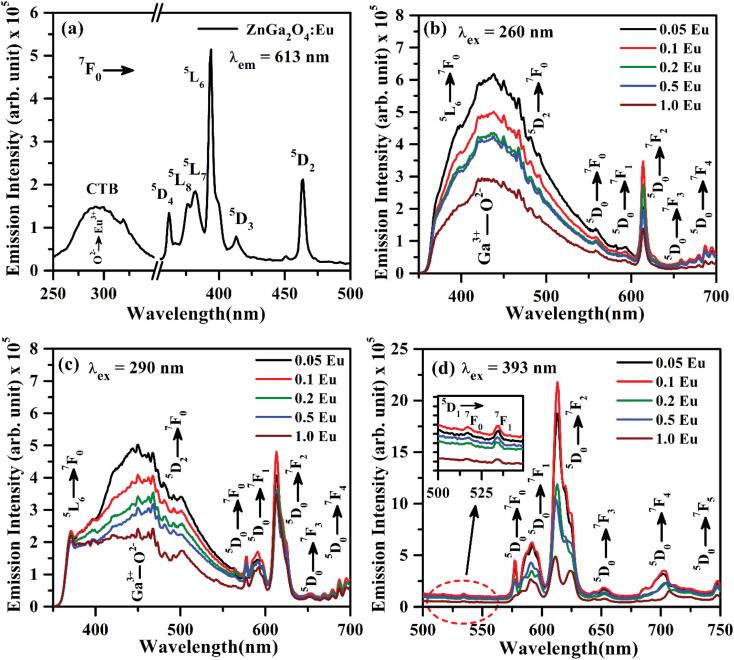
(a) PL excitation spectrum of ZnGa_2_O_4_:0.1Eu^3+^ phosphor with *λ*_em_ = 613 nm, PL emission spectra of ZnGa_2_O_4_:*x*Eu^3+^ phosphors (where *x* = 0.05, 0.1, 0.2, 0.5 and 1.0 mol%) under (b) *λ*_ex_ = 260 nm, (c) *λ*_ex_ = 290 nm and (d) *λ*_ex_ = 393 nm excitation. The inset in (d) is the zoomed PL emission spectra in the 500–549 nm range.

The PL excitation spectrum of ZnGa_2_O_4_:Eu^3+^ phosphor monitored in the 250–500 nm region with *λ*_em_ = 613 nm is shown in Fig. 7(a). The spectrum consists of a broad band ranging from 250–350 nm along with large number of sharp peaks due to intra-configurational forbidden 4f–4f transitions of Eu^3+^ ion.^40–43^ The broad band maxima observed at 290 nm is due to charge transfer band (CTB) of Eu^3+^ ion (O^2−^ → Eu^3+^). The narrow peaks observed at 362, 375, 382, 393, 413 and 463 nm are ascribed to arise due to ^7^F_0_ → ^5^D_4_, ^7^F_0_ → ^5^L_8_, ^7^F_0_ → ^5^L_7_, ^7^F_0_ → ^5^L_6_, ^7^F_0_ → ^5^D_3_ and ^7^F_0_ → ^5^D_2_ transitions of Eu^3+^ ion, respectively.^20–26,40–43^ Among these peaks, the excitation peaks at 393 and 463 nm appear with relatively large intensity.

Fig. 7(b) shows the PL emission spectra of ZnGa_2_O_4_:*x*Eu^3+^ phosphors (where *x* = 0.05, 0.1, 0.2, 0.5 and 1.0 mol%) recorded in 350–700 nm region under the excitation with 260 nm. The spectra show the broad band ranging from 350 to 550 nm due to self-activated emission of the ZnGa_2_O_4_ host with maxima at 434 nm superimposed with Eu^3+^ emission bands in which the bands in higher wavelength side from 550 to 700 nm are very intense. Similar results are also obtained under the excitation with CTB of Eu^3+^ at 290 nm, which is shown in Fig. 7(c). It is clear from the figure that the emission intensity of Eu^3+^ bands is better on excitation with charge transfer band (CTB) at 290 nm as compared to ZnGa_2_O_4_ excitation band at 260 nm. It is interesting to note that the emission peaks due to Eu^3+^ at 393 nm (^5^L_6_ → ^7^F_0_) and 463 nm (^5^D_2_ → ^7^F_0_) transitions are also superposed on the broad emission on excitation with 260 and 290 nm wavelengths.

The intense emission peaks positioned at 577, 592, 613, 652 and 696 nm are attributed to the ^5^D_0_ → ^7^F_0_, ^5^D_0_ → ^7^F_1_, ^5^D_0_ → ^7^F_2_, ^5^D_0_ → ^7^F_3_ and ^5^D_0_ → ^7^F_4_ transitions of Eu^3+^ ion, respectively, which are clearly shown in Fig. 7(b and c).^20–26,40–43^Fig. 7(d) shows the PL emission spectra in the range of 500–750 nm under the excitation at 393 nm. The inset in Fig. 7(d) shows the zoomed emission spectra of Eu^3+^ in the range 500–549 nm. The emission peaks could be marked clearly at 519 and 534 nm due to ^5^D_1_ → ^7^F_0_ and ^5^D_1_ → ^7^F_1_ transitions of Eu^3+^ ion, respectively. The PL emission intensity of Eu^3+^ bands is maximum on excitation with 393 nm as compared to 290 and 260 nm. The band at 613 nm due to ^5^D_0_ → ^7^F_2_ transition exhibits the highest PL emission intensity for all excitation wavelengths. The emission intensity is optimum for 0.1 mol% concentration of Eu^3+^ ion.^44,45^

**Fig. 8:**
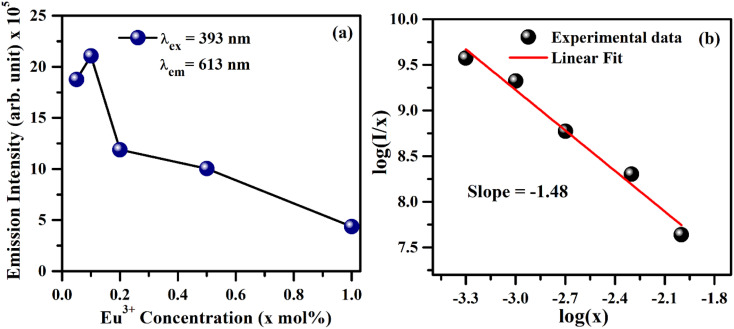
(a) Variation of PL emission intensity *versus* concentration of Eu^3+^ ion in the ZnGa_2_O_4_:*x*Eu^3+^ phosphor samples (where *x* = 0.05, 0.1, 0.2, 0.5 and 1.0 mol%) and (b) a plot between log(*I*/*x*) and log(*x*).

As is seen from Fig. 7, the intensity *I*_^5^D_0_ → ^7^F_2__ ≫ *I*_^5^D_0_ → ^7^F_1__. This clearly shows that the substitution of Eu^3+^ is at asymmetric site in the host lattice. Moreover, it is well known that the ^5^D_0_ → ^7^F_2_ transition of Eu^3+^ ion is due to electric dipole which obeys the selection rule Δ*J* = ±2. Because of the absence of center of symmetry in this host matrix, such transitions are hypersensitive and affected by the local crystal field symmetry around the Eu^3+^ ion. On the other hand, the magnetic dipole transition (^5^D_0_ → ^7^F_1_) follows the selection rule Δ*J* = ±1, and not affected by the local crystal field.^21–27^ The photoluminescence emission intensity of ZnGa_2_O_4_:*x*Eu^3+^ has been monitored for different concentration of Eu^3+^ (where *x* = 0.05, 0.1, 0.2, 0.5 and 1.0 mol%). It is found that the emission intensity increases from 0.05 to 0.1 mol% and then decreases for higher concentrations due to concentration quenching. The variation of Eu^3+^ ion concentration *versus* the emission intensity for 613 nm band under the excitation at 393 nm is shown in Fig. 8(a). The concentration quenching has been observed above 0.1 mol% concentration of Eu^3+^ ion. On increasing the concentration of Eu^3+^ ion, the distance between two Eu^3+^ ions decreases, which increases the mutual interaction between the Eu^3+^ ions due to which the emission intensity of Eu^3+^ band is quenched.

The value of average critical distance between the two Eu^3+^ ions has been calculated using the relation:^16,21,23,41,46,47^vii
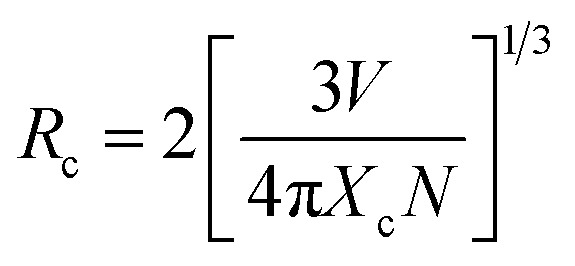
where *V* is the volume of unit cell, *X*_c_ is the critical concentration of Eu^3+^ ion and *N* is the number of Eu^3+^ ions occupying per unit cell in the host lattice. For the cubic spinel crystal structure, *V* = 578.81 Å^3^, *N* = 8 and *X*_c_ = 0.1 respectively.^46^ The calculated value of *R*_c_ using these parameters is found to be 11.13 Å. It is known that when the *R*_c_ value is less than 5 Å, the nature of interaction between two Eu^3+^ ions is exchange interaction. However, in the present case this distance is greater than 5 Å, therefore a multipolar interaction is the main cause of concentration quenching. The exact nature of this multipolar interaction for quenching the PL intensity can be confirmed by the following relation:^16,21,23,41,46,47^viii
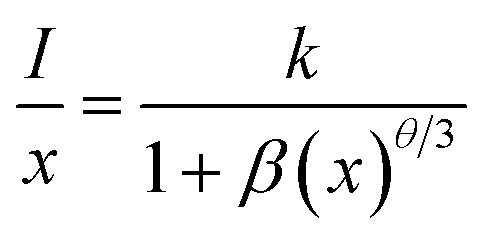
where *I*/*x* is the PL emission intensity per activator concentration. *k* and *β* terms are constants for a given phosphor. The value of *θ* decides the actual nature of interaction between the activator ions. If this value is near to 6, the interaction is dipole–dipole. However, if this value is ∼8 or 10, the nature of interaction will be dipole–quadrupole or quadrupole–quadrupole, respectively. The simplified form of the eqn (viii) can be written as:^21,41,46,47^xi
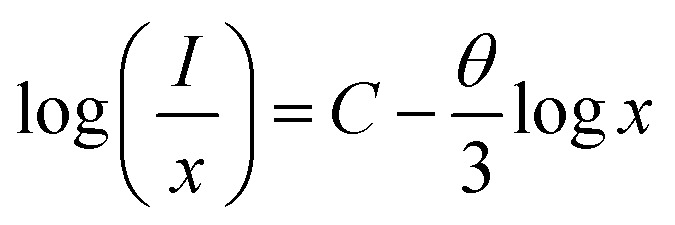
where *C* is a constant. The term −*θ*/3 is the slope of the curve in between 
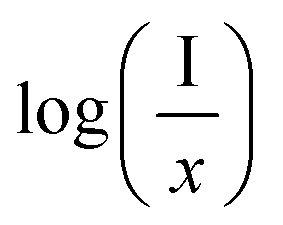
 and log *x*. Fig. 8(b) shows this plot for different concentrations of Eu^3+^ ions for 613 nm under 393 nm excitation in the present case. A linear fit of this gives the slope value −1.48 from which the value of *θ* is found to be 4.44, which is close to 6. This indicates that the dipole–dipole interaction is responsible for quenching of the PL intensity of Eu^3+^ bands in ZnGa_2_O_4_ phosphor.

#### 3.2.3 Effect of Mg^2+^ and Ca^2+^ doping on the PL intensity of ZnGa_2_O_4_:Eu^3+^

**Fig. 9:**
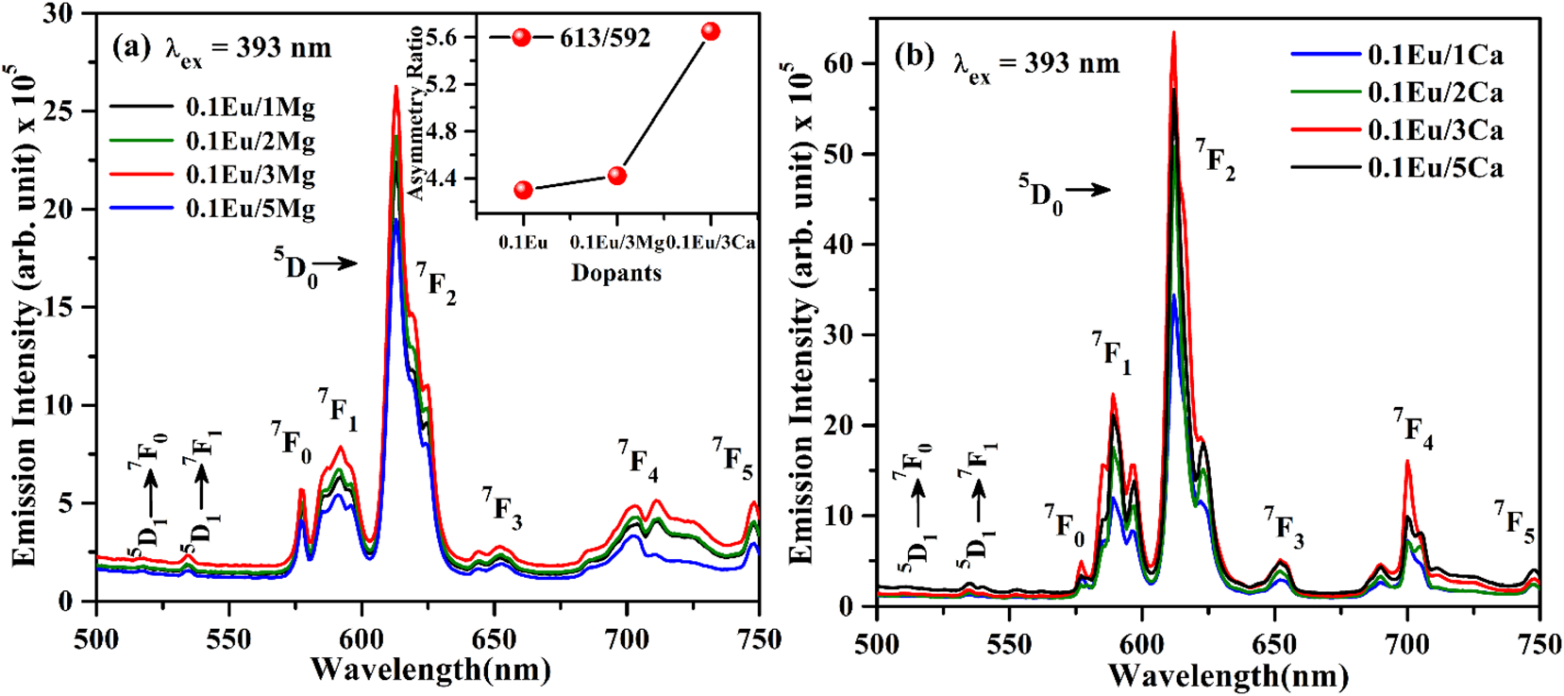
PL emission spectra of (a) ZnGa_2_O_4_:Eu^3+^/*y*Mg^2+^ (b) ZnGa_2_O_4_:Eu^3+^/*z*Ca^2+^ (where *y*/*z* = 1, 2, 3, 5 mol%) with *λ*_ex_ = 393 nm.

The PL emission spectra of ZnGa_2_O_4_:0.1Eu^3+^/*y*Mg^2+^ and ZnGa_2_O_4_:0.1Eu^3+^/*z*Ca^2+^ phosphors (where *y*/*z* = 1, 2, 3 and 5 mol%) monitored on excitation with 393 nm are shown in Fig. 9(a and b). From the Fig. 9(a and b), it is clear that the ZnGa_2_O_4_:0.1Eu^3+^/*y*Mg^2+^ and ZnGa_2_O_4_:0.1Eu^3+^/*z*Ca^2+^ phosphors emit similar emission bands as has been observed in the ZnGa_2_O_4_:0.1Eu^3+^ phosphor. The PL intensity of phosphor samples initially increases up to 3 mol% concentrations of Mg^2+^ and Ca^2+^ ions and then decrease for higher concentrations of these ions. The PL intensity is found to be maximum for 3 mol% concentrations of Mg^2+^/Ca^2+^ ions in the ZnGa_2_O_4_:0.1Eu^3+^ phosphor. The PL intensity of ZnGa_2_O_4_:0.1Eu^3+^ phosphor is increased upto 1.20 and 2.91 times on doping of 3 mol% Mg^2+^ and Ca^2+^ ions, respectively. Thus, the Ca^2+^ doping yields relatively larger PL emission intensity compared to Mg^2+^ in the ZnGa_2_O_4_:0.1Eu^3+^ phosphor. The enhancement in PL intensity of phosphor materials has been reported in several hosts *via* incorporation of Mg^2+^/Ca^2+^ ions.^24,38,48^

The increase in PL intensity of the ZnGa_2_O_4_:0.1Eu^3+^ phosphor *via* Mg^2+^ and Ca^2+^ doping is due to charge imbalance in between the triply ionized Ga and doubly ionized Mg/Ca ions. This causes a crystal field around Eu^3+^ ion, which enhances its emission intensity. Since this field is larger in the case of Ca^2+^ ion than that of Mg^2+^ ion, the enhancement in PL intensity is more in the case of Ca^2+^ doping. The particles size of ZnGa_2_O_4_:0.1Eu^3+^ phosphor is improved from 1.11 to 1.36 and 1.54 μm through Mg^2+^ and Ca^2+^ doping, respectively. The larger particles have large number of activator ions which also contributes to this enhancement. Meetei *et al.* have also observed an enhancement in PL intensity in the YVO_4_:Dy^3+^ phosphor *via* doping of Ca^2+^ ion.^48^

The inset in Fig. 9(a) shows the asymmetry ratio of ZnGa_2_O_4_:0.1Eu^3+^, ZnGa_2_O_4_:0.1Eu^3+^/3Mg^2+^ and ZnGa_2_O_4_:0.1Eu^3+^/3Ca^2+^ phosphor samples upon 393 nm excitation, which clearly demonstrates to the enhancement in PL intensity. The asymmetry ratio of the electric dipole transition *i.e.* (^5^D_0_ → ^7^F_2_) to the magnetic dipole transition *i.e.* (^5^D_0_ → ^7^F_1_) *versus* 0.1 mol% Eu^3+^ doped and 3 mol% Mg^2+^ and Ca^2+^ co-doped phosphor samples for 613 nm emission band, respectively. The asymmetry ratio signifies the nature of crystal field around the Eu^3+^ ion, which is responsible for larger PL intensity. It is clear from the inset of figure that the asymmetry ratio is larger for Ca^2+^ doping compared to Mg^2+^ doping [see Table 2]. The larger value of asymmetry ratio induces larger photoluminescence in the Ca^2+^ co-doped ZnGa_2_O_4_:Eu^3+^ phosphor compared to the Mg^2+^ co-doped ZnGa_2_O_4_:Eu^3+^.

**Table 2:** Asymmetry ratio (*I*_613 nm_/*I*_592 nm_) for ZnGa_2_O_4_:Eu^3+^, ZnGa_2_O_4_:Eu^3+^/3Mg^2+^ and ZnGa_2_O_4_:Eu^3+^/3Ca^2+^ phosphors on excitation with 393 nm

Phosphor	Asymmetric ratio (*I*_613 nm_/*I*_592 nm_)
ZnGa_2_O_4_:0.1Eu^3+^	4.29
ZnGa_2_O_4_:0.1Eu^3+^/3Mg^2+^	4.42
ZnGa_2_O_4_:0.1Eu^3+^/3Ca^2+^	5.65

**Fig. 10:**
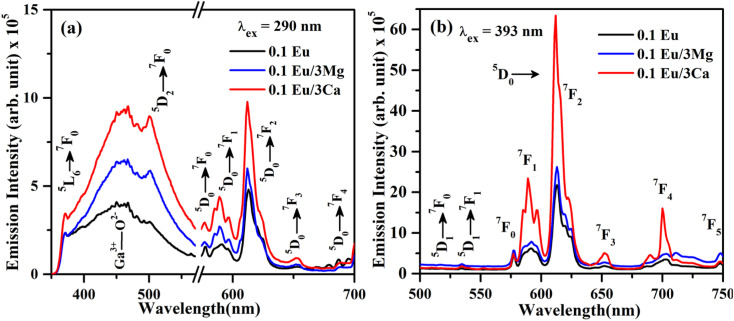
PL emission spectra of ZnGa_2_O_4_:Eu^3+^, ZnGa_2_O_4_:Eu^3+^/3Mg^2+^ and ZnGa_2_O_4_:Eu^3+^/3Ca^2+^ phosphor samples under (a) *λ*_ex_ = 290 nm and (b) *λ*_ex_ = 393 nm excitation.

Fig. 10(a) shows the PL emission spectra of ZnGa_2_O_4_:0.1Eu^3+^, ZnGa_2_O_4_:0.1Eu^3+^/3Mg^2+^ and ZnGa_2_O_4_:0.1Eu^3+^/3Ca^2+^ phosphor samples at *λ*_ex_ = 290 nm in the range 350–700 nm. It is clear from the figure that the PL emission intensity of host as well as of Eu^3+^ bands are enhanced in presence of Mg^2+^ and Ca^2+^ ions. This is due to crystal field of these ions.^23,24,38,47,48^ When these phosphor samples are excited with *λ*_ex_ = 393 nm, the host is not excited. The emission bands are observed only due to Eu^3+^ ion in 500–750 nm range [see Fig. 10(b)]. A similar structure is also obtained in the case of 290 nm excitation; however, the PL intensity is relatively larger for Ca^2+^ doping. The PL intensity of Eu^3+^ band at 613 nm is enhanced upto 1.20 and 2.91 times for Mg^2+^ and Ca^2+^ doping, respectively.

#### 3.2.4 Effect of annealing on the PL intensity

**Fig. 11:**
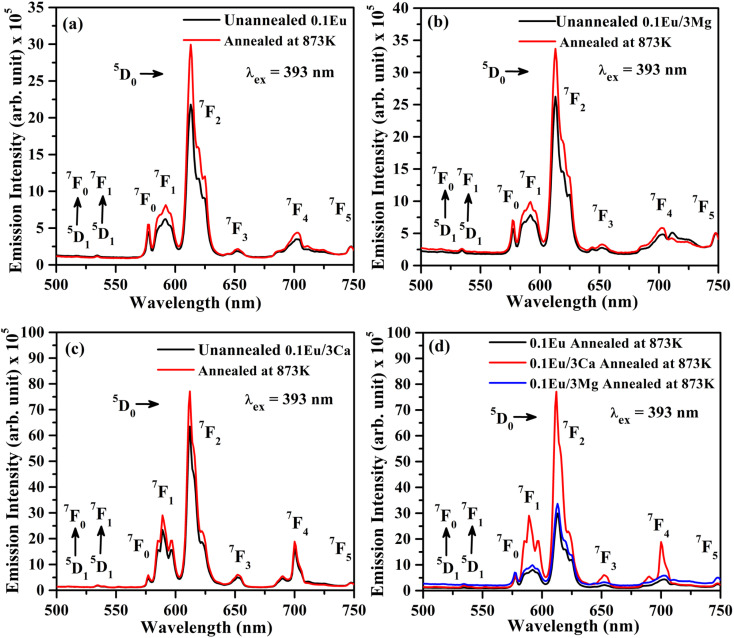
PL emission spectra of unannealed and annealed (at 873 K) (a) ZnGa_2_O_4_:0.1Eu^3+^, (b) ZnGa_2_O_4_:0.1Eu^3+^/3Mg^2+^ and (c) ZnGa_2_O_4_:0.1Eu^3+^/3Ca^2+^ phosphor samples on excitation with *λ*_ex_ = 393 nm. (d) A comparison of the PL intensity of annealed samples at 873 K.

Fig. 11(a–c) shows the PL emission spectra of ZnGa_2_O_4_:0.1Eu^3+^, ZnGa_2_O_4_:0.1Eu^3+^/3Mg^2+^ and ZnGa_2_O_4_:0.1Eu^3+^/3Ca^2+^ phosphor samples unannealed and annealed (at 873 K) for 4 h upon 393 nm excitation. The PL intensity of the samples is found to enhance appreciably for the samples annealed at 873 K temperature. The PL intensity of ZnGa_2_O_4_:0.1Eu^3+^/3Ca^2+^ phosphor is enhanced more than the ZnGa_2_O_4_:0.1Eu^3+^/3Mg^2+^ and ZnGa_2_O_4_:0.1Eu^3+^ phosphor samples.^24,38,47,48^ Actually, the crystallinity of phosphor samples is improved on annealing at 873 K temperature, which affects the PL intensity. Sreena *et al.* have observed an improvement in the PL intensity of phosphor due to increase in crystallinity as the phosphor samples were calcined at 800 and 900 °C temperatures.^49^ Similarly, Kaewnuama *et al.* have also found an increase in PL intensity of the Eu^3+^ doped lithium lanthanum borate phosphor prepared at higher temperatures.^50^ A similar result has been also observed in our case.

In order to understand the change in PL intensity due to annealing, we have also compared the PL intensity of ZnGa_2_O_4_:0.1Eu^3+^, ZnGa_2_O_4_:0.1Eu^3+^/3Mg^2+^ and ZnGa_2_O_4_:0.1Eu^3+^/3Ca^2+^ phosphor samples annealed at 873 K on excitation with 393 nm (see Fig. 11(d)). It is clear from the figure that the PL intensity of ZnGa_2_O_4_:0.1Eu^3+^ phosphor increases in presence of Mg^2+^ and Ca^2+^ ions on annealing.

#### 3.2.5 CIE chromaticity diagram

**Fig. 12:**
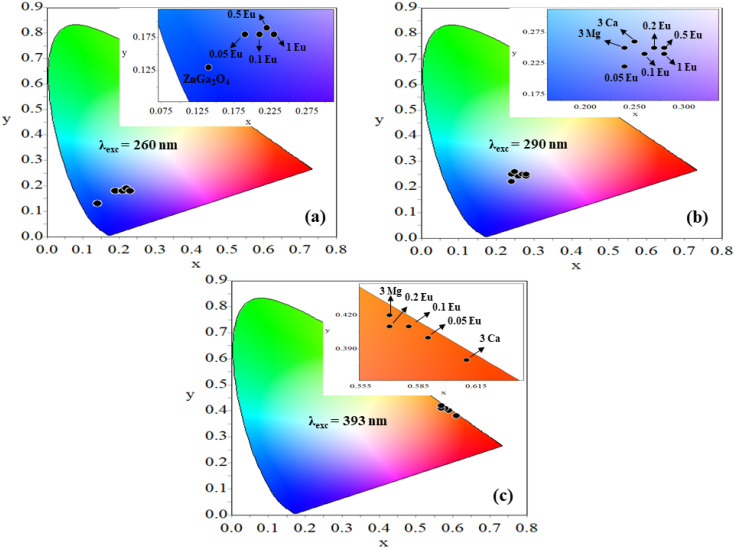
CIE diagrams of (a) ZnGa_2_O_4_ and ZnGa_2_O_4_:*x*Eu^3+^ (where *x* = 0.05, 0.1, 0.2, 0.5 and 1.0 mol%) phosphors on excitation with 260 nm (b) ZnGa_2_O_4_:*x*Eu^3+^, ZnGa_2_O_4_:0.1Eu^3+^/3Mg^2+^ and ZnGa_2_O_4_:0.1Eu^3+^/3Ca^2+^ phosphors on excitation with 290 nm (c) ZnGa_2_O_4_:*x*Eu^3+^, ZnGa_2_O_4_:0.1Eu^3+^/3Mg^2+^ and ZnGa_2_O_4_:0.1Eu^3+^/3Ca^2+^ phosphors on excitation with 393 nm.

The CIE (Commission Internationale de E'clarage) coordinates for the ZnGa_2_O_4_, ZnGa_2_O_4_:*x*Eu^3+^ (where *x* = 0.05, 0.1, 0.2, 0.5 and 1.0 mol%), ZnGa_2_O_4_:0.1Eu^3+^/3Mg^2+^ and ZnGa_2_O_4_:0.1Eu^3+^/3Ca^2+^ phosphors on excitation with 260, 290 and 393 nm wavelengths have been plotted using GoCIE software and they are shown in Fig. 12(a–c). The obtained CIE coordinates for pure ZnGa_2_O_4_ phosphor under 260 nm excitation lies in deep blue region [see Fig. 12(a)]. On doping of different concentrations of *x*Eu^3+^, 0.1Eu^3+^/3Mg^2+^ and 0.1Eu^3+^/3Ca^2+^ in the ZnGa_2_O_4_ phosphor, the CIE coordinates are tuned slightly in the blue region. The coordinates are shifted from pure blue to bluish-white regions on doping of *x*Eu^3+^ and co-doping of 0.1Eu^3+^/3Mg^2+^ and 0.1Eu^3+^/3Ca^2+^ ions in the ZnGa_2_O_4_ phosphor upon 290 nm excitation^35,36^ [see Fig. 12(b)].

Further, the samples glow with bright red color on excitation with 393 nm, the CIE coordinates varying in the red region for different concentrations of Eu^3+^ ions [see Fig. 12(c)]. On co-doping of 3Mg^2+^ and 3Ca^2+^ ions in the ZnGa_2_O_4_:0.1Eu^3+^ phosphor, the CIE coordinates shift from (0.57, 0.42) to (0.61, 0.38). The CIE coordinates (0.61, 0.38), are close to the National Television System Committee (NTSC) standard value for a pure red color (0.67, 0.33). From this, it is clear that the co-doping of Mg^2+^ and Ca^2+^ ions in the ZnGa_2_O_4_:Eu^3+^ phosphor not only enhances the emission intensity but also improves the color perception. This shows that the emitted color is tunable with excitation wavelengths, which are useful in display devices. The calculated values of CIE coordinates are given in Table 3.

**Table 3:** CIE coordinates for the ZnGa_2_O_4_, ZnGa_2_O_4_:0.1Eu^3+^, ZnGa_2_O_4_:0.1Eu^3+^/3Mg^2+^ and ZnGa_2_O_4_:0.1Eu^3+^/3Ca^2+^ phosphors under 260, 290 and 393 nm excitations

Phosphor	CIE coordinates (*x*,*y*) at *λ*_ex_ = 260 nm	CIE coordinates (*x*,*y*) at *λ*_ex_ = 290 nm	CIE coordinates (*x*,*y*) at *λ*_ex_ = 393 nm
ZnGa_2_O_4_	(0.14,0.13)		
ZnGa_2_O_4_:0.05Eu^3+^	(0.19,0.18)	(0.24,0.22)	(0.59,0.40)
ZnGa_2_O_4_:0.1Eu^3+^	(0.21,0.18)	(0.26,0.24)	(0.58,0.41)
ZnGa_2_O_4_:0.2Eu^3+^	(0.22,0.19)	(0.27,0.25)	(0.57,0.41)
ZnGa_2_O_4_:0.5Eu^3+^	(0.22,0.19)	(0.28,0.24)	(0.57,0.42)
ZnGa_2_O_4_:1.0Eu^3+^	(0.23,0.18)	(0.28,0.25)	(0.57,0.42)
ZnGa_2_O_4_:0.1Eu^3+^/3Mg^2+^		(0.24,0.25)	(0.57,0.42)
ZnGa_2_O_4_:0.1Eu^3+^/3Ca^2+^		(0.25,0.26)	(0.61,0.38)

The color purity of the phosphor samples has been calculated by using the following relation.^36^x
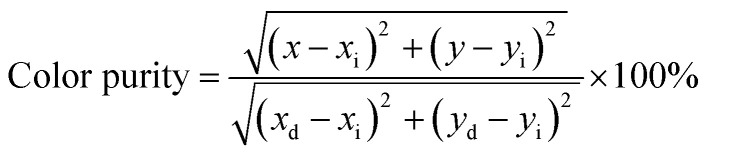
where (*x*,*y*), (*x*_i_,*y*_i_) and (*x*_d_,*y*_d_) are the CIE coordinates of phosphor, the standard light source for white light and the used dominant wavelength, respectively. The value of color purity of pure ZnGa_2_O_4_ is 85.87%. However, the color purity of the ZnGa_2_O_4_:0.1Eu^3+^ was calculated to 93.62% upon 393 nm excitation.

#### 3.2.6 Lifetime measurements

**Fig. 13:**
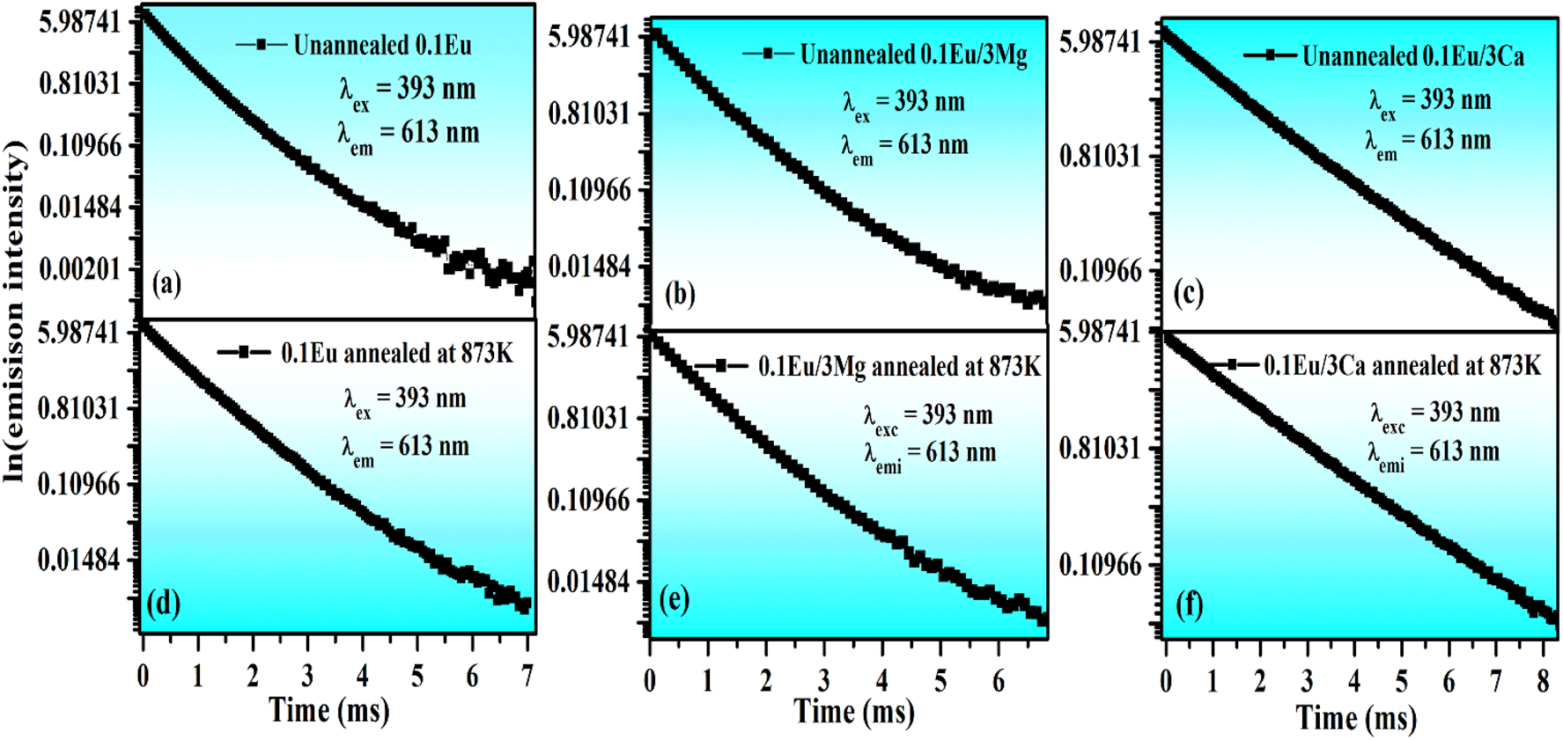
Decay curves of unannealed (a) ZnGa_2_O_4_:0.1Eu^3+^ (b) ZnGa_2_O_4_:0.1Eu^3+^/3Mg^2+^ and (c) ZnGa_2_O_4_:0.1Eu^3+^/3Ca^2+^ phosphor samples and of the annealed (d) ZnGa_2_O_4_:0.1Eu^3+^ (e) ZnGa_2_O_4_:0.1Eu^3+^/3Mg^2+^ and (f) ZnGa_2_O_4_:0.1Eu^3+^/3Ca^2+^ phosphor on excitation with 393 nm.

Fig. 13(a–f) shows the decay curves for the (^5^D_0_ → ^7^F_2_) transition at 613 nm for ZnGa_2_O_4_:0.1Eu^3+^, ZnGa_2_O_4_:0.1Eu^3+^/3Mg^2+^ and ZnGa_2_O_4_:0.1Eu^3+^/3Ca^2+^ phosphor samples unannealed and annealed (at 873 K) on excitation with 393 nm, respectively. The decay curves were found to fit better using a bi-exponential relation.^16^xi
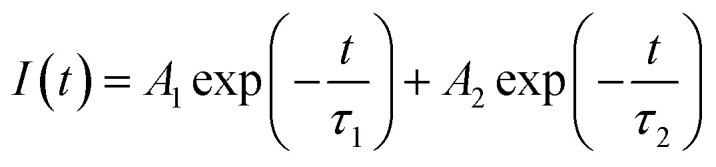
where *τ*_1_ and *τ*_2_ are the larger and smaller values of decay times and other terms have their usual meaning. The average lifetime values were calculated using the relation.^38^xii
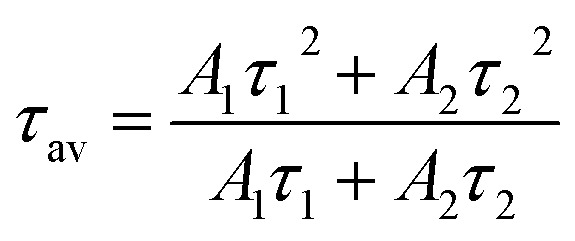


The average value of lifetime for ZnGa_2_O_4_:0.1Eu^3+^ phosphor is found to be 0.69 ms and for the same sample annealed at 873 K; it is found as 0.71 ms. On co-doping of Mg^2+^ and Ca^2+^ ions in the ZnGa_2_O_4_:0.1Eu^3+^ phosphor, the lifetime values were found to be 0.73 and 1.51 ms. When these samples were annealed at 873 K, the values of average lifetime were found to be 0.78 and 1.65 ms. From this, it is clear that the lifetime value is increased on co-doping of Mg^2+^ and Ca^2+^ ions in the ZnGa_2_O_4_:Eu^3+^ phosphor sample and it is further increased in case of annealed samples. This is due to highly crystalline nature of materials with less surface defects in the case of annealed phosphor samples.

#### 3.2.7 Temperature dependent photoluminescence of ZnGa_2_O_4_:0.1Eu^3+^ and ZnGa_2_O_4_:0.1Eu^3+^/3Ca^2+^ phosphors and thermal stability

**Fig. 14:**
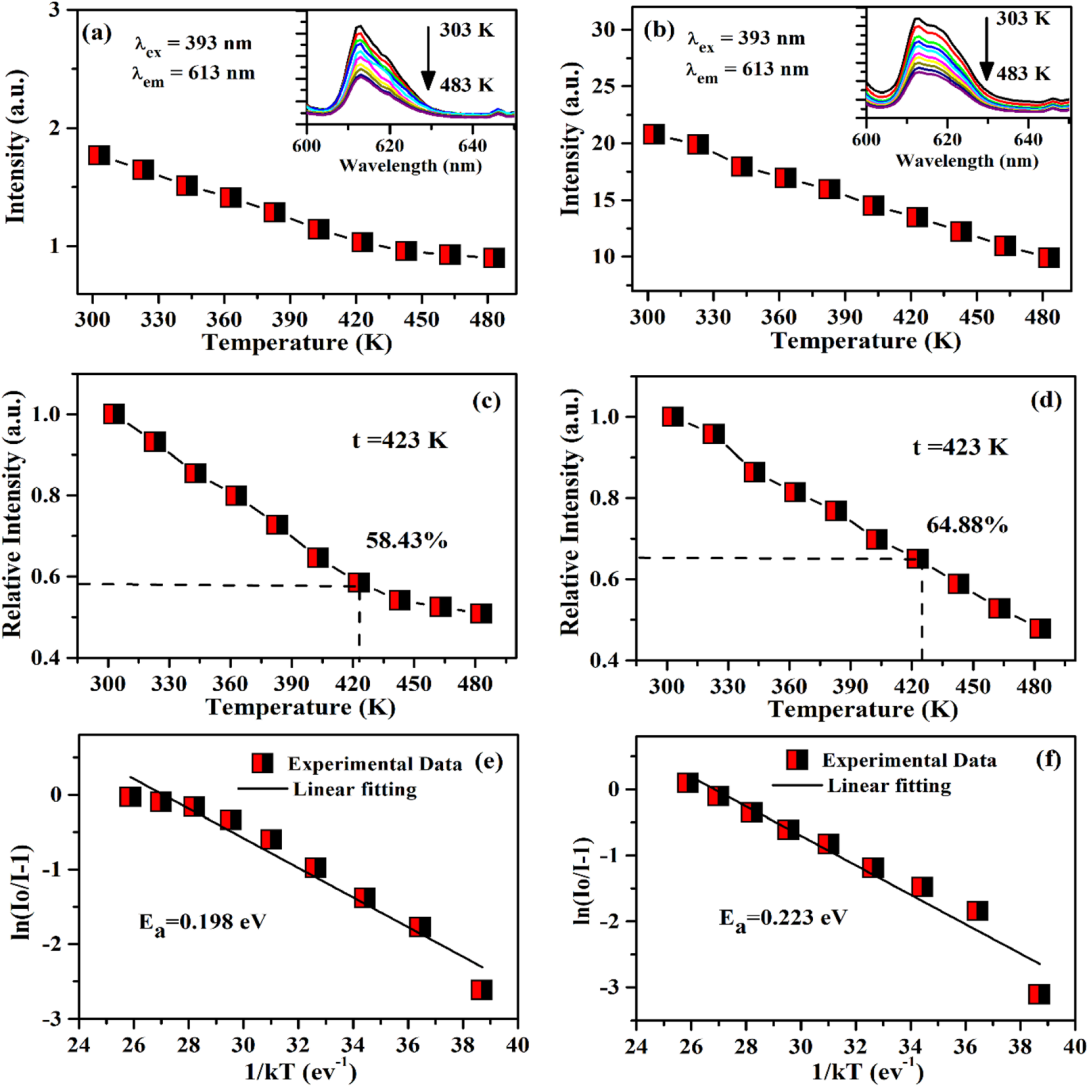
Variation in PL emission intensity *versus* temperature for 613 nm band of (a) ZnGa_2_O_4_:0.1Eu^3+^ and (b) ZnGa_2_O_4_:0.1Eu^3+^/3Ca^2+^ phosphor samples. Relative PL intensity at different temperatures for (c) ZnGa_2_O_4_:0.1Eu^3+^ and (d) ZnGa_2_O_4_:0.1Eu^3+^/3Ca^2+^ phosphor samples. Arrhenius plot ln[(*I*_0_/*I*) − 1] *versus* 1/*kT* for (e) ZnGa_2_O_4_:0.1Eu^3+^ and (f) ZnGa_2_O_4_:0.1Eu^3+^/3Ca^2+^ phosphor samples under the excitation at 393 nm.

In order to analyze the thermal stability of the phosphor samples, we have monitored the temperature dependent PL emission spectra of ZnGa_2_O_4_:0.1Eu^3+^ and ZnGa_2_O_4_:0.1Eu^3+^/3Ca^2+^ phosphor samples in the temperatures range 303–483 K under 393 nm excitation. As it is clear from Fig. 14(a and b), the PL emission intensity of Eu^3+^ bands reduces continuously with the rise in temperature due to thermal quenching effect.^29–33^ The emission intensity of 613 nm emission band is reduced upto 58.43% and 64.88% at 423 K for the ZnGa_2_O_4_:0.1Eu^3+^ [see Fig. 14(c)] and ZnGa_2_O_4_:0.1Eu^3+^/3Ca^2+^ [see Fig. 14(d)], respectively as compared to their initial intensity at room temperature (303 K). From this, it is clear that the doping of Ca^2+^ ion in the ZnGa_2_O_4_:0.1Eu^3+^ phosphor exhibits better thermal stability as compared to the ZnGa_2_O_4_:0.1Eu^3+^ phosphor. We have also calculated the activation energy as it is an important parameter to characterize the thermal stability of the phosphor materials. It is well known that for better thermal stability of the phosphor materials, the activation energy should have larger value. The activation energy of phosphor samples has been also calculated using Arrhenius equation:^51,52^xiii
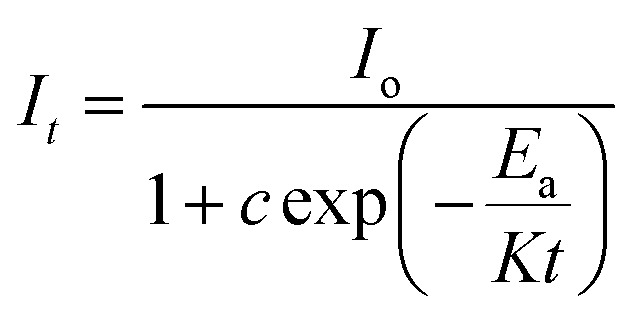
where *I*_o_ and *I*_*t*_ are the emission intensities of the phosphor samples at room temperature and at temperature (*t*). The term *E*_a_ is the activation energy and *K* is the Boltzmann's constant. The activation energy has been calculated by plotting the graph in between ln[(*I*_0_/*I*) − 1] *vs.* 1/*kT* as shown in Fig. 14(e and f). The slope of these curves gives the values of the activation energy 0.198 eV (see Fig. 14(e)) and 0.223 eV (see Fig. 14(f)) for the ZnGa_2_O_4_:0.1Eu^3+^ and ZnGa_2_O_4_:0.1Eu^3+^/3Ca^2+^ phosphor samples, respectively. Zhang *et al.* have obtained the value of activation energy 0.17 eV for the Eu^3+^ doped BaZrGe_3_O_9_ phosphor.^51^ The activation energy has been also found 0.14 eV in the case of Eu^3+^ doped Ca_19_Mg_2_(PO_4_)_14_ phosphor.^52^ It has been observed that the activation energy is much larger for the Eu^3+^ doped and Eu^3+^/Ca^2+^ co-doped ZnGa_2_O_4_ phosphor samples. This reveals that the Eu^3+^ doped and Eu^3+^/Ca^2+^ co-doped ZnGa_2_O_4_ phosphors show better thermal stability. Moreover, the thermal stability is increased in presence of Ca^2+^ ion. The Ca^2+^ co-doping plays an important role to reduce the influence of non-radiative relaxations.

**Fig. 15:**
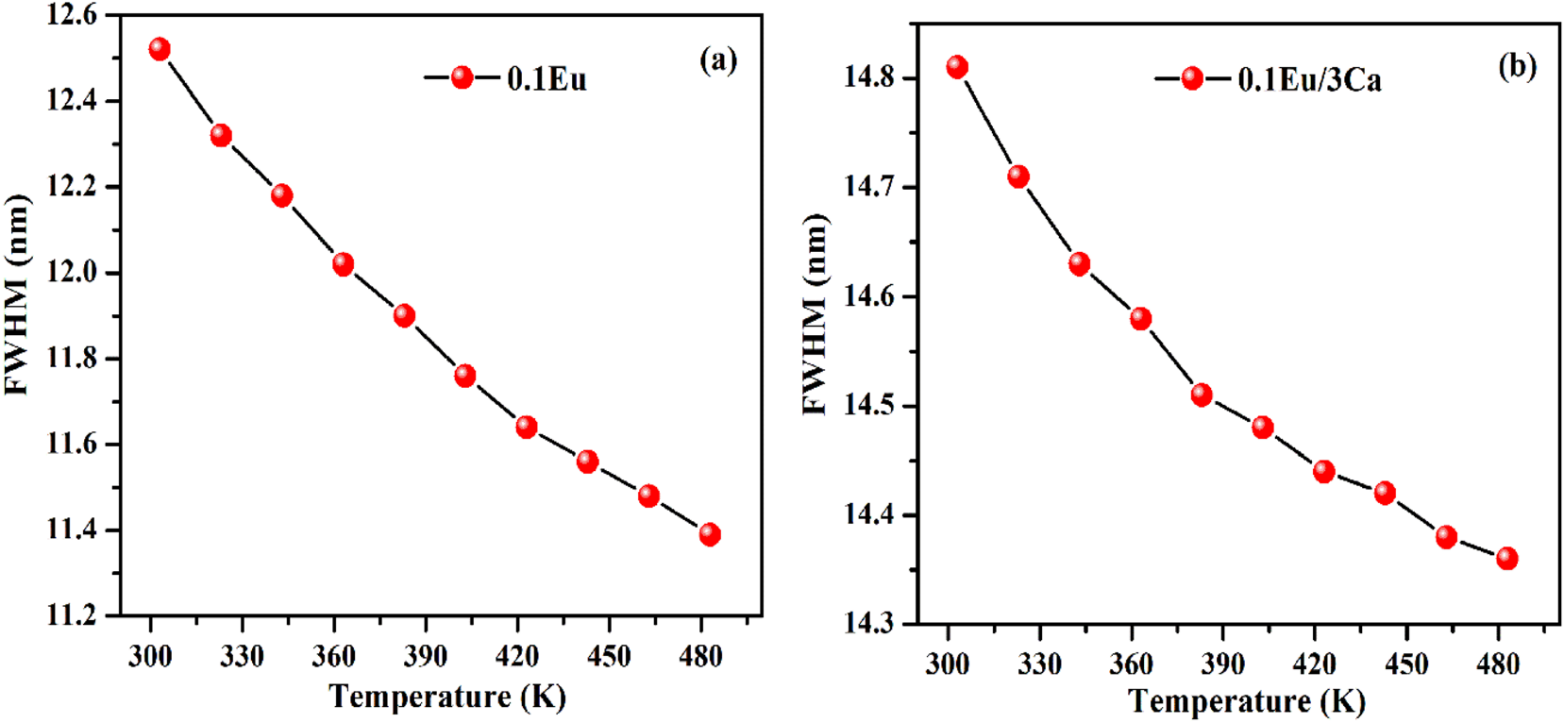
(a and b) Variation in FWHM of 613 nm peak for the ZnGa_2_O_4_:0.1Eu^3+^ and ZnGa_2_O_4_:0.1Eu^3+^/3Ca^2+^ phosphor samples with temperature.

It is clear from insets of Fig. 14(a and b) that the FWHM of 613 nm peak in the ZnGa_2_O_4_:0.1Eu^3+^ and ZnGa_2_O_4_:0.1Eu^3+^/3Ca^2+^ phosphor decreases continuously on increasing the temperature of these samples. The FWHM of the 613 nm peak in the case of ZnGa_2_O_4_:0.1Eu^3+^ phosphor decreases rapidly while in the case of ZnGa_2_O_4_:0.1Eu^3+^/3Ca^2+^ phosphor it decreases slowly.^29^Fig. 15(a and b) shows the variation in FWHM of the peaks in the ZnGa_2_O_4_:0.1Eu^3+^ and ZnGa_2_O_4_:0.1Eu^3+^/3Ca^2+^ phosphors as a function of temperature supplied to the samples. This reveals that the ZnGa_2_O_4_:0.1Eu^3+^/3Ca^2+^ phosphor is more thermally stable than the ZnGa_2_O_4_:0.1Eu^3+^ phosphor. Thus, the doping of Ca^2+^ in ZnGa_2_O_4_:0.1Eu^3+^ enhances the stability of the phosphor material.

## 4. Conclusions

The Eu^3+^ doped and Mg^2+^/Ca^2+^ co-doped ZnGa_2_O_4_ phosphor samples were successfully synthesized by solid-state reaction method at 1473 K. A small part of all the samples were further annealed for four hours at 873 K, which improves the PL intensity of the phosphor samples. The XRD, SEM, EDS, FTIR spectra of all the samples were monitored. The pure ZnGa_2_O_4_ emits broad blue emission (350–550 nm) on UV (260 nm) excitation. However, Eu^3+^ doped and Mg^2+^/Ca^2+^ co-doped samples emit blue and red bands on 260 and 290 nm excitations. However, excitation of doped samples with 393 nm emits intense pure red color. The PL intensity of emission bands is enhanced up to 1.20 and 2.91 times *via* co-doping of Mg^2+^ and Ca^2+^ ions in the Eu^3+^ doped phosphors due to increase in the crystallinity, particles size and crystal field due to charge imbalance. The blue emission due to pure ZnGa_2_O_4_ sample shifts slowly towards the bluish-white and red regions for Eu^3+^ doped and Ca^2+^/Mg^2+^ co-doped ZnGa_2_O_4_ phosphors on excitation with 290 and 393 nm wavelengths. For 393 nm excitation, it emits only red in all cases. The lifetime of ^5^D_0_ level of Eu^3+^ ion increases on Ca^2+^/Mg^2+^ doping and on annealing the sample. The temperature dependent photoluminescence (TDPL) study shows the thermal quenching behavior of the sample with thermal stability ∼65% and activation energy of 0.223 eV in the Eu^3+^/Ca^2+^ co-doped phosphor. Thus, the Eu^3+^ doped and Ca^2+^ co-doped ZnGa_2_O_4_ phosphor is highly thermally stable under external temperature, which may be useful for display devices, blue LEDs, warm red LEDs and plant growth lighting applications.

## Conflicts of interest

Authors declare that there is no conflict of interest in the present study.

## Supplementary Material
